# Efficacy and safety of shensong yangxin capsules for persistent atrial fibrillation: a Systematic Review and meta-analysis of randomized controlled trials

**DOI:** 10.3389/fphar.2025.1620340

**Published:** 2025-06-18

**Authors:** Yifan Chen, Liuding Wang, Yuying Li, Runa A, Jiahui OuYang, Zhonghui Jiang, Zhuye Gao

**Affiliations:** ^1^ Department of Cardiology, Xiyuan Hospital, China Academy of Chinese Medical Sciences, Beijing, China; ^2^ Department of Neurology, Xiyuan Hospital, China Academy of Chinese Medical Sciences, Beijing, China; ^3^ College of Basic Medicine, Capital Medical University, Beijing, China; ^4^ Department of Internal Medicine, Yanke Hospital, China Academy of Chinese Medical Sciences, Beijing, China

**Keywords:** persistent atrial fibrillation, Shensong Yangxin capsule, meta-analysis, Systematic review, traditional Chinese medicine

## Abstract

**Background:**

Shensong Yangxin (SSYX), a standardized Chinese preparation, is widely utilized in arrhythmia treatment. This research sought to assess the clinical advantages of SSYX for persistent atrial fibrillation (PsAF).

**Methods:**

We searched seven databases and two registries to identify randomized controlled trials (RCTs) assessing SSYX as an adjunctive treatment for PsAF. We assessed methodological quality with the Cochrane Risk of Bias Tool 2.0, and conducted meta-analyses with RevMan 5.4.

**Results:**

The meta-analysis incorporated ten RCTs enrolling 1,713 patients with PsAF. SSYX combined with conventional treatments (CTs) significantly lowered the recurrence of AF compared to CTs alone (risk ratio [*RR*] = 0.65, 95% conffdence interval [*CI*] 0.56 to 0.75, *P* < 0.001). The results also showed that SSYX contributed to the reduction of left atrial diameter (*MD* = −1.41, 95% *CI* -2.48 to −0.34, *P* < 0.001) and P-wave dispersion (*MD* = −10.37, 95% *CI* -17.23 to −3.5, *P* = 0.003). Safety analysis revealed that the combination of SSYX and CTs decreased adverse reaction incidence (*RR* = 0.54, 95% *CI* 0.32 to 0.90, *P* = 0.02). The certainty of evidence was graded as moderate to low.

**Conclusion:**

SSYX showed potential in preventing AF recurrence in PsAF patients. Nevertheless, these preliminary findings require validation through more rigorously designed trials, given methodological limitations impacting evidence certainty.

**Systematic review registration:**

https://www.crd.york.ac.uk/PROSPERO/view/CRD420251008974, identifier CRD420251008974.

## Introduction

Atrial fibrillation (AF) has shown a significant rise in both prevalence and incidence globally (Kornej et al., 2020; Brundel et al., 2022). Epidemiological research revealed that AF had affected approximately 33 million people worldwide (Chung et al., 2020; Li et al., 2022). By 2030, the number of individuals with AF in the United States is projected to reach 12.1 million, with 2.6 million new cases (Joglar et al., 2024). Approximately 70% of patients with AF are diagnosed with persistent AF (PsAF), which is defined as episodes lasting over 7 days or long-standing cases lasting over 12 months (Zoni-Berisso et al., 2014). Compared with paroxysmal AF, the persistent subtype shows a stronger association with higher AF burden and increased cardiovascular risks, such as stroke and all-cause mortality (Steinberg et al., 2015). Rhythm control serves as the cornerstone of the comprehensive management of AF (Blomström-Lundqvist et al., 2019). Currently, the first-line therapeutic approaches for PsAF mainly consist of catheter ablation and anti-arrhythmic drugs (AADs) (Van Gelder et al., 2024). However, even after undergoing catheter ablation, patients with PsAF have a remarkably high recurrence rate of AF, up to 46.2% within 1 year after the operation (Crowley et al., 2024). Findings from the CABANA trial showed that 19.4% of patients who had catheter ablation needed repeated ablation because of AF recurrence (Packer et al., 2019). This repeated procedure increased the risk of adverse events, such as pulmonary vein stenosis and stiff left atrial syndrome (Schoene et al., 2019). After ablation, short-term administration of AADs significantly lowers the risk of early recurrence of atrial arrhythmias; however, it has little effect on lowering the risk of late recurrence. High discontinuation rates indicate that side effects or lack of efficacy could hinder their long-term use (Arbelo et al., 2017). In conclusion, continuous exploration and optimization of rhythm control strategies for PsAF are essential.

Traditional Chinese medicines (TCMs) have shown substantial potential in controlling arrhythmia (Li et al., 2024; Ge et al., 2025). Shensong Yangxin (SSYX), a Chinese patent medicine for arrhythmia, enhances qi, nourishes yin, and improves blood circulation to clear collaterals. The main components are *Panax ginseng* C.A.Mey. (Ren Shen), *Paeonia lactiflora* Pall. (Chi Shao), *Ophiopogon japonicus* (Thunb.) Ker Gawl. (Mai Dong), *Taxillus chinensis* Danse (Sang Ji Sheng), *Eupolyphaga sinensis* Walker (Tu Bie Chong), *Cornus officinalis* Siebold & Zucc. (Shan Zhu Yu), *Coptis chinensis* Franch. (Huang Lian), *Polypodium pseudo-amoenum* Ching (Long Gu), *Ziziphus jujuba* Mill. (Suan Zao Ren), *Salvia miltiorrhiza* Bunge. (Dan Shen), *Schisandra sphenanthera* Rehder and E.H. Wilson (Wu Wei Zi), and *Nardostachys grandiflora* DC. (Gan Song). In China, SSYX is recommended for AF treatment in various guidelines (Medicines, 2021; Zhang et al., 2021; Association et al., 2022). Previous clinical studies (Tuo et al., 2020; He et al., 2024) indicated that SSYX helped maintain sinus rhythm and improved cardiac function. Experiments (Yang et al., 2022; Zhang J. et al., 2023) have revealed that SSYX inhibits electrical and structural remodeling, reduces intracellular iron overload, suppresses inflammatory responses and oxidative stress, and protects endothelial cell function. These effects are crucial for regulating heart rhythm and enhancing cardiac function.

Recent years have seen the emergence of multiple randomized controlled trials (RCTs) investigating SSYX for PsAF treatment. Therefore, our aim was to assess the efficacy and safety of SSYX as an adjuvant treatment for PsAF, with a focus on the recurrence of AF.

## Methods

### Protocol register

The research protocol was prospectively registered in PROSPERO (CRD420251008974). Methodological implementation strictly complied with the PRISMA 2020 statement (Page et al., 2021).

### Search strategy

We conducted a comprehensive search of PubMed, EMBASE, the Cochrane Library, China National Knowledge Infrastructure, Wanfang, China Science and Technology Journal Database, and Chinese Biomedical Literature Database up to 20 March 2025. Regarding unpublished trials, we also performed a thorough search on ClinicalTrials.gov and the Chinese Clinical Trial Registry. Supplementary Table S1 outlines the comprehensive database search strategies.

On 25 April 2025, we refreshed our search by applying the same search approach, limiting it to articles published subsequent to 20 March 2025.

### Inclusion criteria

Inclusion criteria included: 1) Study design: RCTs. 2) Population: Patients diagnosed with PsAF (Van Gelder et al., 2024). 3) Intervention: The intervention group received SSYX treatment on the basis of conventional treatments (CTs), while the control group were allocated to CTs alone or CTs plus placebo. CTs encompassed rhythm control (catheter ablation and AADs), rate control, and anticoagulation (Chinese Society of Cardiology and Engineering, 2023; Van Gelder et al., 2024). 4) Outcomes: The primary outcome was recurrence of AF (the occurrence of confirmed atrial tachyarrhythmia lasting ≥30s). The secondary outcomes were left atrial diameter (LAD), P-wave dispersion (Pd), left ventricular ejection fraction (LVEF), left ventricular end-diastolic diameter (LVEDD), N-terminal pro-B-type natriuretic peptide (NT-proBNP), and high-sensitivity C-reactive protein (hs-CRP). Each trial must report at least one of the specified outcomes mentioned above. Safety outcomes were measured by the incidence of adverse events and adverse reactions.

### Exclusion criteria

Exclusion criteria included: 1) Suspected duplicate trials presenting concordant data; 2) Trials with incongruent data and conclusions, thereby causing apprehensions regarding academic integrity; 3) Trials in which the treatment is combined with herbal prescriptions other than SSYX.

### Study selection

The initial stage of literature screening involved using NoteExpress 3.2 software to remove duplicates. Yifan Chen and Liuding Wang independently evaluated the articles by reviewing their titles, abstracts, and full texts. Any disagreement was addressed via consultations with the third reviewer (Zhonghui Jiang).

### Data extraction

Two researchers extracted data and conducted cross-checks. The information included the design of the study, populations, treatments, and outcomes. The numbers of participants and events were documented for dichotomous outcomes. The number of participants, the mean values, and the standard deviations were noted down for continuous outcomes. In the case of trials that provided quartiles, the method by Tong et al. was used to determine the data skewness and calculate the mean and standard deviation (Wan et al., 2014; Luo et al., 2018).

### Risk of bias assessment

Two researchers employed the Cochrane Risk of Bias Tool 2.0 to evaluate the trial methodology. The aspects evaluated encompassed the randomization process, blinding, data integrity, and inadequate findings publication (Sterne et al., 2019). Discrepancies were settled via discussion with the third author.

### Data analysis

Meta-analyses were conducted with the RevMan 5.4. A fixed-effects model was employed when there was insignificant heterogeneity (*I* ^*2*^ ≤ 50%), whereas a random-effects model was adopted in the case of substantial heterogeneity (*I* ^*2*^ > 50%). The risk ratio (RR) was employed to evaluate dichotomous outcomes. For continuous outcomes, we computed the weighted mean difference (MD) or the standardized MD, both presented with a 95% confidence interval (CI). A p-value less than 0.05 indicated statistical significance. Subgroup analyses were conducted to assess the influence of clinical heterogeneity, including variations in follow-up duration, on the aggregated results. Although initially planned, the assessment of publication bias was not performed. This was due to the tests’ unreliability when <10 studies are available for a specific outcome.

### Certainty of evidence

We evaluated the evidence for outcomes using the Grading of Recommendations Assessment, Development and Evaluation (GRADE) approach (Balshem et al., 2011).

## Results

### Study selection

**FIGURE 1:**
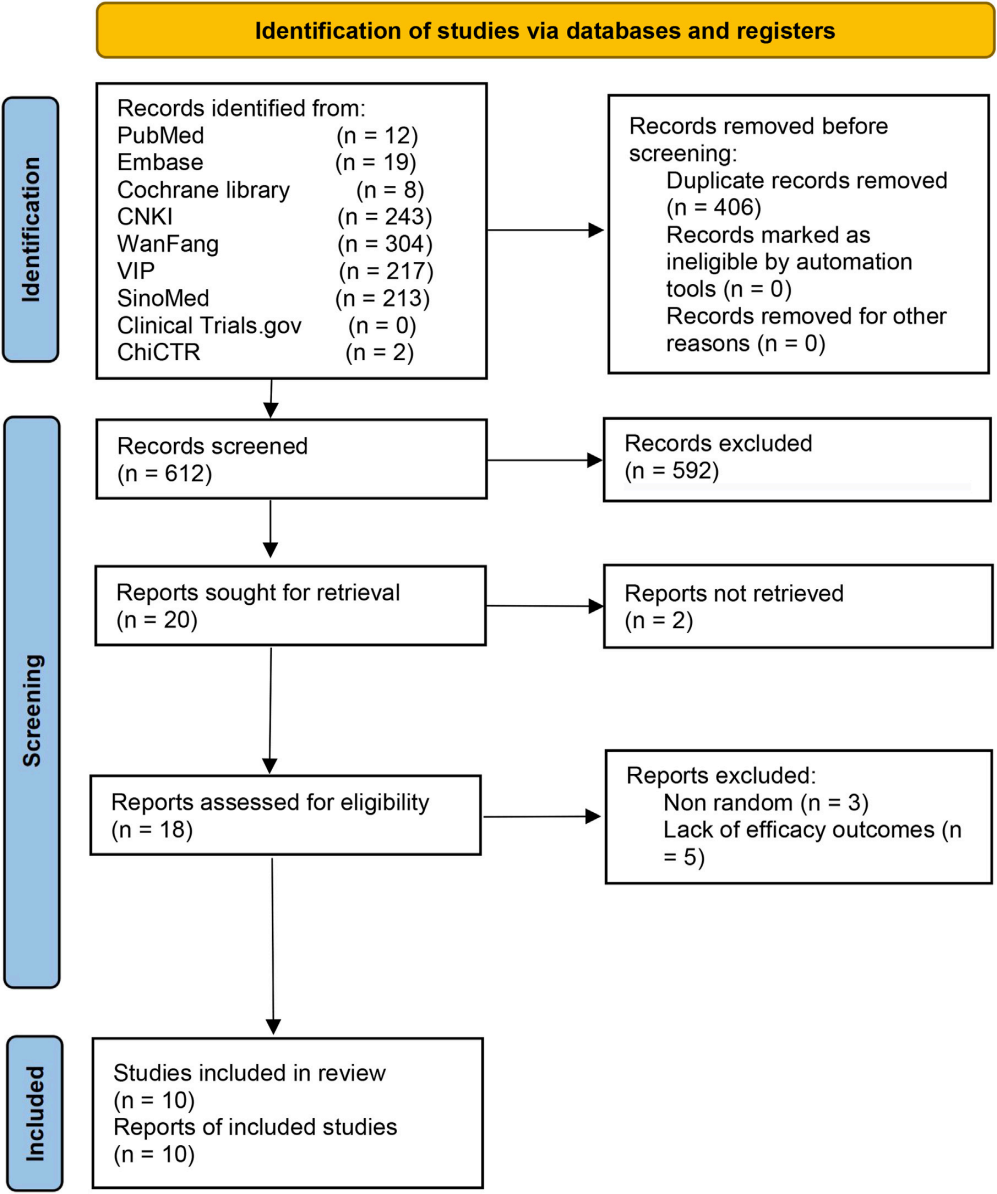
The Preferred Reporting Items for Systematic Reviews and Meta-Analyses flow diagram for study selection.

A total of 1,018 records were retrieved (Figure 1). After deleting 406 duplicate records, we excluded 592 ineligible records by screening the titles and abstracts. Apart from the two records reported as inaccessible, we reviewed full-texts of the remaining 18 trials. We excluded eight trials for reasons including non-randomized and absence of efficacy outcomes (Supplementary Table S2). Finally, ten studies were included (Du, 2013; Yu and Liu, 2014; Jin, 2017; Zhuo et al., 2017; He et al., 2018; Zhou et al., 2019; Li, 2020; Zhou and Lu, 2021; Zhang G. W. et al., 2023; Huang et al., 2024).

### Study characteristics

Ten included RCTs involved 1,713 participants. The minimum sample size was 60, and the maximum was 882. A total of 862 participants received SSYX plus CTs, while 851 received CTs or placebo plus CTs. There was a multi-center trial led by Renmin Hospital of Wuhan University and conducted across 66 clinical centers (Huang et al., 2024). The remaining trials were single-center RCTs carried out at nine Class-A tertiary hospitals. These hospitals are situated in Rizhao, Weihai, Tai’an, and Jinan (Shandong Province), Shanghai, Wuhan (Hubei Province), Fuzhou (Jiangxi Province) and Anyang (Henan Province) across East and Central China.

On the basis of the identical CTs, a single RCT conducted a comparison between SSYX and a placebo, while nine RCTs carried out comparisons of SSYX against no intervention. CTs encompassed rhythm control (catheter ablation and AADs), rate control, and anticoagulation. The treatment duration of the included trials ranged from two to 12 months. Six RCTs reported recurrence of AF (Yu and Liu, 2014; Jin, 2017; Zhuo et al., 2017; Zhou et al., 2019; Zhang G. W. et al., 2023; Huang et al., 2024). The demographic baselines, interventions, and outcomes are presented in Table 1. Supplementary Table S3 details the source, quality control, and chemical characteristics of SSYX used in the trials.

**TABLE 1:** Overview of study characteristics.

Study	Sample size		Male/Famale		Age/(year)		Intervention		Duration/(month)	Outcomes
	T	C	T	C	T	C	T	C		
Huang et al. (2024)	443	439	342/101	304/135	62 (54, 68)	63 (55, 68)	(1) SSYX 1.6g three times daily (2) CTs including radiofrequency ablation, anticoagulant, and AADs used during the blank period	(1) CTs (2) Placebo	12	①③④⑤⑧⑨
Zhang et al. (2023a)	30	30	23/7	23/7	61.57 ± 10.23	62.22 ± 11.93	(1) SSYX 1.6g three times daily (2) CTs including radiofrequency ablation, anticoagulant, and AADs	(1) CTs	6	①③④⑨
Zhou and Lu (2021)	42	42	18/24	17/25	61.25 ± 5.39	61.32 ± 5.42	(1) SSYX 1.6g three times daily (2) CTs including valsartan 80 mg daily, captopril 25 mg daily, anticoagulant, and AADs	(1) CTs	3	②⑨
Li (2020)	49	48	22/27	20/28	59.03 ± 5.26	58.91 ± 5.19	(1) SSYX 0.8g–1.6g three times daily (2) CTs including valsartan 80 mg daily	(1) CTs	3	③⑤
Zhou et al. (2019)	60	60	35/25	33/27	56.28 ± 4.58	57.45 ± 4.45	(1) SSYX 1.6g three times daily (2) CTs including radiofrequency ablation, AADs, and anticoagulant. Amiodarone was administered at a dosage of 0.6 g daily for 1 week, then adjusted to 0.4 g daily for 1 week, and finally reduced to 0.2 g daily thereafter. Meanwhile, the administration of warfarin was adjusted according to the INR value ranging from 2 to 3	(1) CTs	3	①③④
He et al. (2018)	43	37	23/20	21/16	61.54 ± 6.32	60.13 ± 7.51	(1) SSYX 1.6g three times daily (2) CTs including valsartan capsules 80 mg daily, anticoagulant, and AADs	(1) CTs	3	③⑤⑨
Zhuo et al. (2017)	40	40	23/17	22/18	56.0 ± 4.8	57.5 ± 5.0	(1) SSYX 1.6g three times daily (2) CTs including radiofrequency ablation, AADs, and anticoagulant. Amiodarone was administered at a dosage of 0.6 g daily for 1 week, then adjusted to 0.4 g daily for 1 week, and finally reduced to 0.2 g daily thereafter. Meanwhile, the administration of warfarin was adjusted according to the INR value ranging from 2 to 3	(1) CTs	6	①③⑤⑥
Jin (2017)	33	33	18/15	19/14	49.9 ± 15.7	48.9 ± 16.2	(1) SSYX 1.6g three times daily (2) CTs including radiofrequency ablation and AADs	(1) CTs	12	①⑦⑨
Yu and Liu (2014)	76	76	43/33	41/35	39-71	40-72	(1) SSYX 1.6g three times daily (2) CTs including radiofrequency ablation, AADs, anticoagulant, and anti-atrial remodeling. Amiodarone 0.2 g daily. Warfarin to keep the INR between 1.5 and 2.5. Irbesartan 150 mg daily	(1) CTs	12	①⑥⑦
Du (2013)	46	46	27/19	25/21	64 ± 5	63 ± 4	(1) SSYX 1.6g three times daily (2) CTs including aspirin, digitalis, β-Blockers etc.	(1) CTs	2	②⑥

Note: AADs, anti-arrhythmic drugs; CTs, conventional treatments; C, control group; INR, international normalized ratio; SSYX, shensong yangxin; T, intervention group; ①, recurrence of atrial fibrillation; ②, P-wave dispersion; ③, left atrial diameter; ④, left ventricular ejection fraction; ⑤, left ventricular end-diastolic diameter; ⑥, N-terminal pro-B-type natriuretic peptide; ⑦, high-sensitivity C-reactive protein; ⑧, adverse events; ⑨, adverse reactions. The data in the age section are presented as median (lower quartile, upper quartile), mean ± standard deviation, or minimum - maximum.

### Methodological quality

**FIGURE 2:**
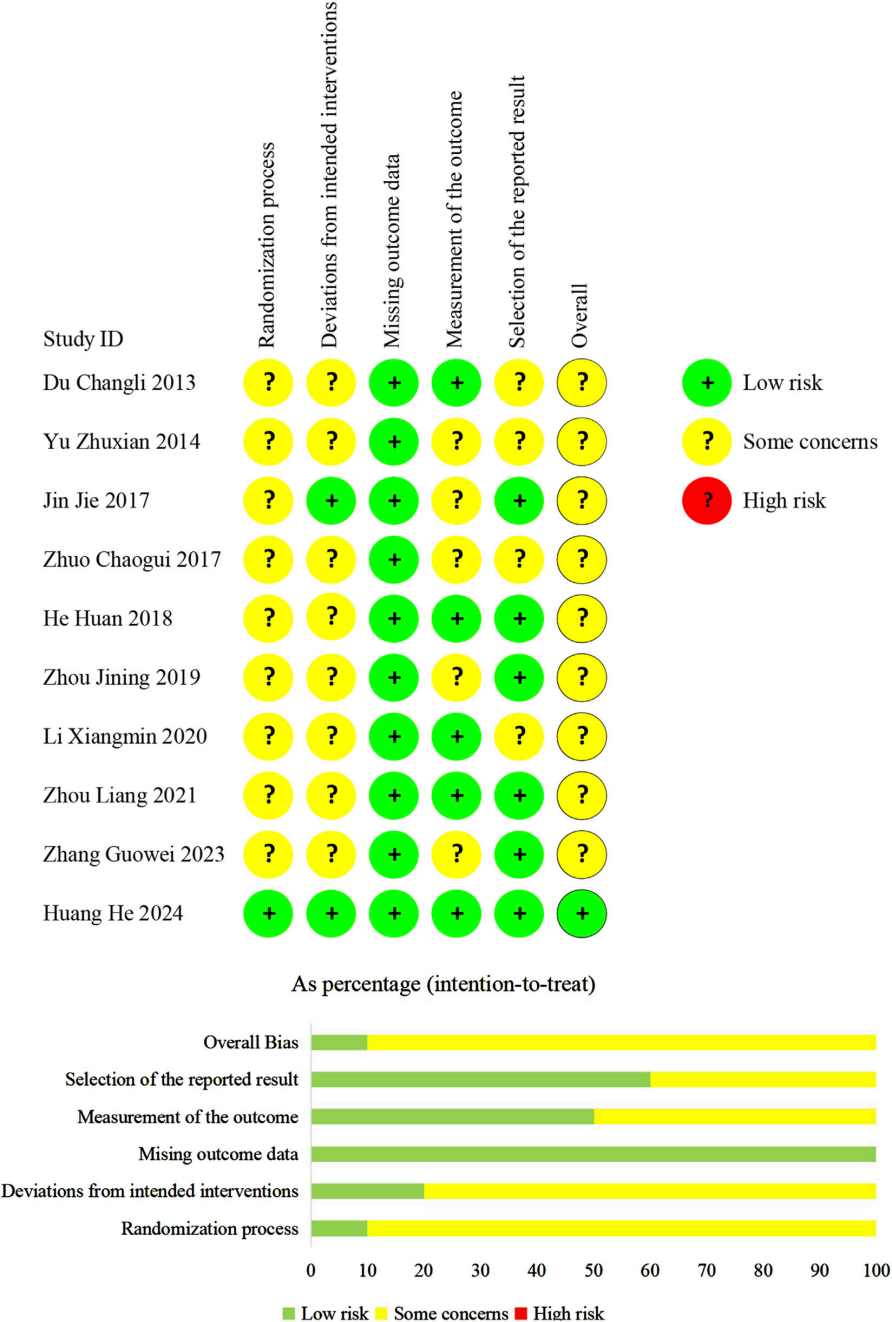
Risk of bias of included studies.

We evaluated the overall bias of one trial as being at a low-risk level. For the remaining trials, the overall bias was considered to have some concerns (Figure 2).

In terms of randomization, one trial (Huang et al., 2024) employed a central system. The system automatically linked each assigned random number to the package number of the corresponding assigned treatments. This guaranteed the concealment of random sequences. We judged the trial (Huang et al., 2024) to have a “low risk of bias.” Six trials (Du, 2013; Yu and Liu, 2014; Jin, 2017; He et al., 2018; Zhou and Lu, 2021; Zhang G. W. et al., 2023) employed random number tables while three trials (Zhuo et al., 2017; Zhou et al., 2019; Li, 2020) did not specify their randomization methods. Allocation concealment details were omitted in all nine trials. Therefore, we judged them to have “some concerns of bias.”

Concerning the deviations from the intended interventions, In a trial (Huang et al., 2024), a placebo was used to blind patients, caregivers, and physicians, addressing deviations from intended interventions. A modified intention-to-treat (ITT) approach (Association et al., 2022) was adopted in this trial, and we considered it to have a “low risk of bias.” Although one trial (Jin, 2017) did not report the blinding method, but it confirmed no deviations from the planned intervention. Therefore, this trial was also evaluated as having a “low risk of bias.” The remaining trials did not use a placebo and reported no information regarding deviations from the intended intervention, but they adopted the ITT analysis. Consequently, we judged them to have “some concerns of bias.”

We judged all trials to have a “low risk of bias” regarding missing outcome data, as data for almost all randomized patients were included in the ITT or modified ITT analyses.

Regarding the measurement of outcomes, only one trial (Huang et al., 2024) mentioned the blinding of assessors, so we judged the trial to have a “low risk of bias.” Although no other trials reported blinding of assessors, four trials (Du, 2013; He et al., 2018; Li, 2020; Zhou and Lu, 2021) reported echocardiographic parameters such as LAD and LVEF, electrocardiographic parameter Pd, or hematological indicators such as NT-proBNP. These outcomes are objective, unaffected by patients’ subjective feelings and observers’ judgments. Therefore, we assessed these four studies as having a “low risk of bias.” The remaining five trials (Yu and Liu, 2014; Jin, 2017; Zhuo et al., 2017; Zhou et al., 2019; Zhang G. W. et al., 2023) reported recurrence of AF, which relied on the observers’ subjective judgments and may be influenced by the observers’ knowledge of the intervention. So, we judged these trials to have “some concerns of bias.”

Regarding the selection of reported results, we assessed six trials (Jin, 2017; He et al., 2018; Zhou et al., 2019; Zhou and Lu, 2021; Zhang G. W. et al., 2023; Huang et al., 2024) to have a “low risk of bias.” This is because they comprehensively reported the predefined outcome measures. In other trials, adverse reactions were not not referred to. These trials were categorized as “some concerns of bias”.

### Primary outcomes

#### Recurrence of AF

**FIGURE 3:**
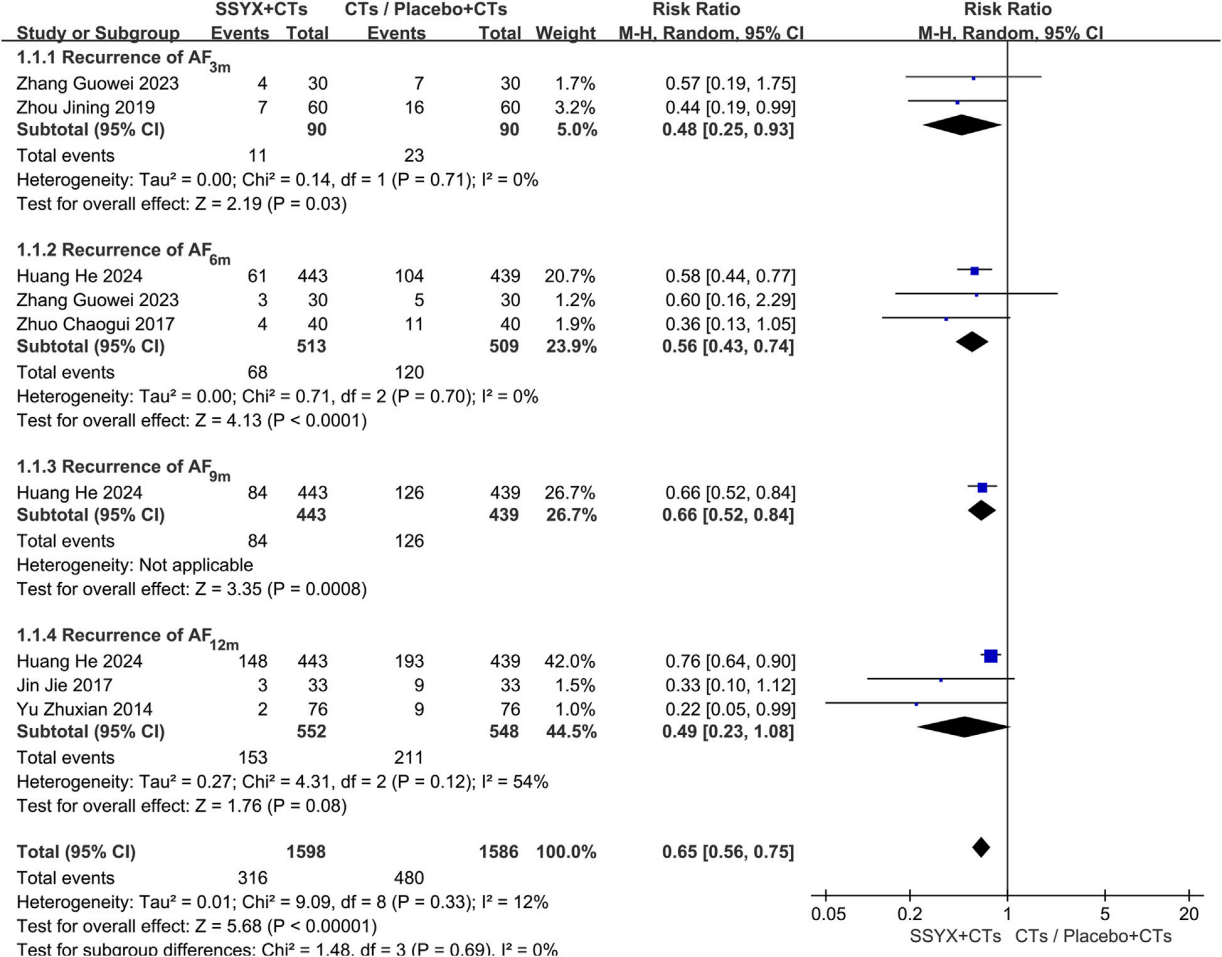
Recurrence of AF: SSYX plus CTs vs. Placebo plus CTs/CTs.

Six trials reported the recurrence of AF at 3 months (Zhou et al., 2019; Zhang G. W. et al., 2023), 6 months (Zhuo et al., 2017; Zhang G. W. et al., 2023), 9 months (Huang et al., 2024), and 12 months (Yu and Liu, 2014; Jin, 2017; Huang et al., 2024). To address clinical heterogeneity, subgroup analyses were conducted according to the follow-up time. The subgroup analysis at 12 months showed significant heterogeneity (*P* = 0.12, *I* ^*2*^ = 54%); therefore, a random-effects model was adopted. The findings demonstrated that SSYX combined with CTs significantly outperformed both placebo with CTs and CTs alone in lowering AF recurrence (*RR* = 0.65, 95% *CI* 0.56 to 0.75, *P* < 0.001; Figure 3). The forest plot showed that the statistical heterogeneity at 12 months mainly stemmed from the differences between one trial (Huang et al., 2024) and the other two trials (Yu and Liu, 2014; Jin, 2017). Specifically, compared with Huang et al.’s trial, the other two trials had smaller sample sizes and did not adopt a placebo - controlled design. These methodological differences might be the main reasons for the statistical heterogeneity.

### Secondary outcomes

#### LAD

Six trials reported the LAD after different durations of SSYX administration: 3 months (He et al., 2018; Zhou et al., 2019; Li, 2020; Zhang G. W. et al., 2023), 6 months (Zhuo et al., 2017; Zhang G. W. et al., 2023), and 12 months (Huang et al., 2024). The combined analysis showed that SSYX with CTs was significantly outperformed placebo with CTs or CTs alone in decreasing LAD (*MD* = −1.82, 95% *CI* -2.94 to −0.71, *P* = 0.001; Supplementary Figure S1). The 6-month subgroup analysis showed no significant heterogeneity (*P* = 0.42, *I* ^*2*^ = 0%), suggesting a steady improvement in LAD after 6-month SSYX administration (*MD*_LAD-6m_ = −1.33, 95% *CI* -2.14 to −0.53, *P* = 0.001; Supplementary Figure S1).

#### Pd

Two trials reported the Pd after different durations of SSYX administration: 2 months (Du, 2013) and 3 months (Zhou and Lu, 2021). The pooled results showed that SSYX with CTs significantly outperformed CTs alone in decreasing Pd (*MD* = −10.37, 95% *CI* -17.23 to −3.5, *P* = 0.003; Supplementary Figure S2).

#### LVEF

Four trials (Zhuo et al., 2017; Zhou et al., 2019; Zhang G. W. et al., 2023; Huang et al., 2024) reported the LVEF. The combined analysis indicated no significant difference in LVEF improvement between the SSYX and the control groups (*MD* = 1.14, 95% *CI* = −0.47 to 2.76, *P* = 0.16; Supplementary Figure S3).

#### LVEDD

LVEDD was reported in four trials (Zhuo et al., 2017; He et al., 2018; Li, 2020; Huang et al., 2024). The combined analysis showed that there was no statistically significant difference in the improvement of LVEDD between the SSYX and the control groups (*MD* = −2.37, 95% *CI* = −5.00 to 0.26, *P* = 0.08; Supplementary Figure S4).

#### NT-proBNP

NT-proBNP was reported in three trials (Du, 2013; Yu and Liu, 2014; Zhuo et al., 2017). The combined treatment of SSYX and CTs was significantly better than CTs alone in loweing the NT-proBNP level (*MD* = −131.87, 95% *CI* = −232.36 to −31.38, *P* = 0.01; Supplementary Figure S5).

#### Hs-CRP

The levels of hs-CRP following 12 months of SSYX treatment were reported in two trials (Yu and Liu, 2014; Jin, 2017). Combined analysis showed that, in comparison with the control group, the hs-CRP levels in the SSYX group were significantly decreased (*MD* = −2.32, 95% *CI* = −2.67 to −1.97, *P* < 0.001; Supplementary Figure S6).

### Safety outcomes

#### Adverse events

**FIGURE 4:**
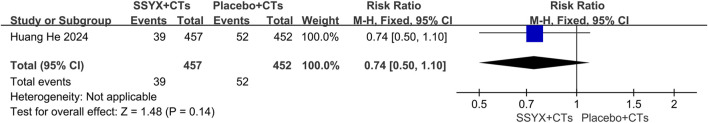
Adverse events: SSYX plus CTs vs. Placebo plus CTs/CTs.

One multicenter trial (Huang et al., 2024) reported serious adverse events. The SSYX group (n = 457) experienced 39 serious adverse events, compared to 52 in the control group (n = 452). Regarding the incidence of serious adverse events, no significant difference was observed between the two groups (*RR*=0.74, 95% *CI* = 0.50 to 1.10, *P* = 0.14; Figure 4).

#### Adverse reactions

**FIGURE 5:**
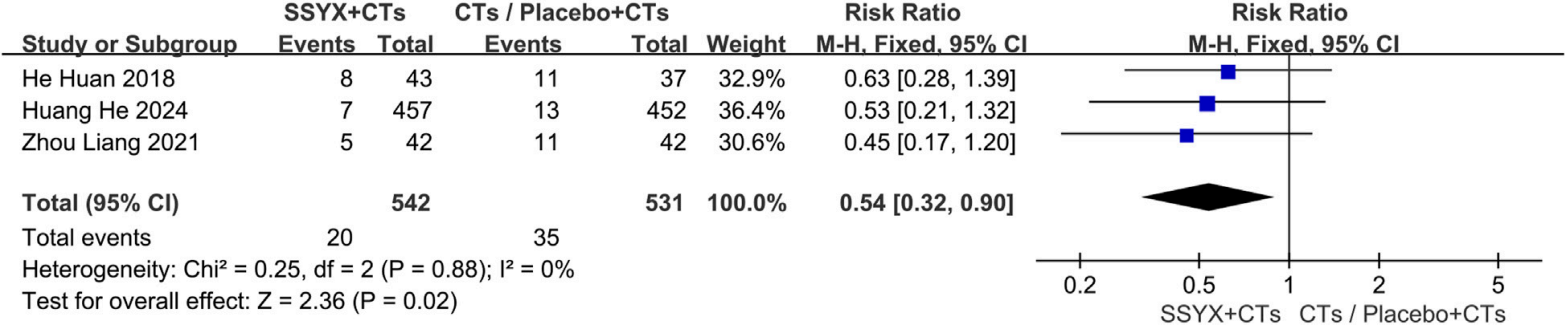
Adverse reactions: SSYX plus CTs vs.Placebo plus CTs/CTs.

No adverse reactions were observed in three trials (Jin, 2017; Zhou et al., 2019; Zhang G. W. et al., 2023). In other studies (He et al., 2018; Zhou and Lu, 2021; Huang et al., 2024), adverse reactions occurred in 20 cases within the SSYX group (n = 542) and 35 cases in the control group (n = 531). Compared with the control group, the incidence of adverse reactions in the SSYX group was significantly lower (*RR* = 0.54, 95% *CI* = 0.32 to 0.90, *P* = 0.02; Figure 5). Gastrointestinal diseases and dizziness were the most frequently occurring adverse reactions in the SSYX group.

#### Certainty assessment

Employing the GRADE approach (Table 2), we determined that the level of evidence varied from moderate to low. This was mainly because of the high risk of bias, imprecision, and heterogeneity.

**TABLE 2:** Certainty of evidence for SYYX in the treatment of patients with PsAF.

Outcomes	No. of participants (studies)	Anticipated Absolute effects (95% *CI*)		Relative effect (95% *CI*)	Certainty of the Evidence
		Risk with CG	Risk difference with, E.G.,		
Recurrence of AF	1,360 (6)	303 per 1,000	350 fewer per 1,000 (440 fewer to 250 fewer)	*RR* 0.65 (0.56–0.75)	⊕⊕⊕○ MODERATE^a^
Recurrence of AF-3m	180 (2)	256 per 1,000	133 fewer per 1,000 (192 fewer to 18 fewer)	*RR* 0.48 (0.25–0.93)	⊕⊕○○ LOW^a,b^
Recurrence of AF-6m	1,022 (3)	236 per 1,000	104 fewer per 1,000 (135 fewer to 61 fewer)	*RR* 0.56 (0.43–0.74)	⊕⊕⊕○ MODERATE^a^
Recurrence of AF-9m	882 (1)	287 per 1,000	98 fewer per 1,000 (138 fewer to 46 fewer)	*RR* 0.66 (0.52–0.84)	⊕⊕⊕○ MODERATE^c^
Serious adverse events	909 (1)	115 per 1,000	30 fewer per 1,000 (58 fewer to 12 more)	*RR* 0.74 (0.50–1.10)	⊕⊕⊕○ MODERATE^c^
Adverse reactions	1,073 (3)	66 per 1,000	30 fewer per 1,000 (45 fewer to 7 fewer)	*RR* 0.54 (0.32–0.90)	⊕⊕⊕○ MODERATE^a^

Abbreviation: CG, control group; *CI*, confidence interval; E.G., experimental group; AF, atrial fibrillation; *RR*, relative risks; Notes: a, poor methodology, including randomization and blinding methods; b, small sample size; c, data provided by only one study; d, heterogeneity with *I* ^*2*^ ≥ 50%.

## Discussion

### Summary of findings

This research centered on assessing the effectiveness of SSYX in lowering the recurrence of AF among patients with PsAF. The findings indicated that the application of SSYX led to a decline in the AF recurrence rate, and this outcome was backed by evidence of moderate certainty. Given its well-defined concept, objective evaluation, and intuitive reflection of treatment effects, the AF recurrence rate is commonly used to evaluate the regulatory effects of rhythm-control therapies on atrial electrical activity (Andrade et al., 2021; Turagam et al., 2023). Moreover, we evaluated LAD, Pd, LVEF, LVEDD, and NT-proBNP, which demonstrated the protective effect of SSYX on cardiac structure and function. LAD is the most commonly used indicator for assessing atrial structure and is linked to the long-term prognosis of AF patients (Menichelli et al., 2021). Studies indicated a positive correlation between an increased LAD and the incidence of heart failure and major adverse cardiac events in AF patients (Froehlich et al., 2019). Pd reflects the heterogeneity of atrial electrical activity and predicts the triggering and recurrence of AF (Chen et al., 2022; Karanikola et al., 2025). In patients with cryptogenic stroke who had an implantable loop recorder, a Pd of 40 milliseconds was the sole independent predictor of AF (Marks et al., 2021).

Recent studies have shown that the anti-arrhythmic effect of SSYX may be achieved through multiple mechanisms. SSYX can enhances iron transporters expression, mitigates intracellular iron overload, and reduces reactive oxygen species production, which collectively inhibit electrical and structural remodeling, thus decreasing AF susceptibility (Yang et al., 2022). SSYX mitigates atrial electrical remodeling by modulating autonomic nerve activity imbalance, enhancing vagal nerve function, regulating various ion channels, extending action potential duration, and alleviating calcium overload (Zhao et al., 2016; Zhao et al., 2017; Yang et al., 2020). Moreover, SSYX can activate endothelial nitric oxide synthase to release nitric oxide to protects endothelial cells (Jiang et al., 2021); reduce type I and type III collagens expression to inhibit myocardial fibrosis (Shen et al., 2014; Ma et al., 2018); and lowers the monocyte chemoattractant protein-1 and inflammatory factors like tumor necrosis factor-α and interleukin-6, exerting an anti-inflammatory effect (Zhang J. et al., 2023). The findings of our review demonstrated that SSYX reduced the hs-CRP level in patients with PsAF, indicating its anti-inflammatory properties.

In terms of SSYX’s safety, the group taking SSYX had fewer serious adverse events, such as heart failure, and a lower incidence of adverse reactions, including gastrointestinal issues and dizziness, compared to the control group. This finding affirms the safety of SSYX treatment.

### Limitations

There were several limitations of the included trials. To start with, the studies only included the Chinese population. This could restrict the applicability of the findings. Further investigation is required to assess how variations in physical traits, familial support, and social beliefs across different ethnic groups or regions may influence the effectiveness of SSYX. Secondly, several included studies failed to report serious adverse events and reactions related to the administration of SSYX. To minimize reporting bias, it is essential to thoroughly and accurately document adverse events and drug-related adverse reactions. Finally, some trials did not employ blinding methods, resulting in flaws in methodology and reducing the certainty of evidence. Most studies have not fully described the random sequence generation and allocation concealment, which may introduce implementation and measurement biases. In future RCTs, it is crucial to strictly apply the allocation concealment method and rigorously implement the double-blind method.

### Implications for future research

In future research, we recommend broadening the scope of the study population and enhancing the methodological quality. International collaboration should be strengthened to facilitate the implementation of more high - quality, international multicenter RCTs regarding SSYX to verify the universality.

Improving the methodological quality involves several key aspects. Firstly, it is essential to emphasize allocation concealment. This measure helps to achieve proper randomization and effectively control selection bias. Secondly, the implementation of double-blinding is crucial. Double-blinding can manage both implementation bias (related to how the treatment is administered) and measurement bias (associated with the assessment of outcomes). Finally, providing detailed reports of adverse events and their associations with the utilization of SSYX is necessary to manage reporting bias.

## Conclusion

Our study highlights the potential of SSYX as an adjuvant treatment for PsAF. Moderate evidence suggests it may reduce recurrence of AF, but additional research is necessary to verify its long-term effectiveness and safety.

## Data Availability

The original contributions presented in the study are included in the article/Supplementary Material, further inquiries can be directed to the corresponding authors.

## Abbreviations

AADs, anti-arrhythmic drugs; AF, atrial fibrillation; CI, confidence interval; CTs, conventional treatments; GRADE, Grading of Recommendations Assessment, Development and Evaluation; hs-CRP, high-sensitivity C-reactive protein; ITT, intention-to-treat; LAD, left atrial diameter; LVEDD, left ventricular end-diastolic diameter; LVEF, left ventricular ejection fraction; MD, mean difference; NT-proBNP, N-terminal pro-B-type natriuretic peptide; Pd, p-wave dispersion; PsAF, persistent atrial fibrillation; RCTs, randomized controlled trials; RR, relative risks; SSYX, Shensong Yangxin; TCMs, traditional Chinese medicines.

## References

[B1] Andrade J. G. Wells G. A. Deyell M. W. Bennett M. Essebag V. Champagne J. (2021). Cryoablation or drug therapy for initial treatment of atrial fibrillation. N. Engl. J. Med. 384 (4), 305–315. 10.1056/NEJMoa2029980 33197159

[B2] Arbelo E. Brugada J. Blomström-Lundqvist C. Laroche C. Kautzner J. Pokushalov E. (2017). Contemporary management of patients undergoing atrial fibrillation ablation: in-hospital and 1-year follow-up findings from the ESC-EHRA atrial fibrillation ablation long-term registry. Eur. Heart J. 38 (17), 1303–1316. 10.1093/eurheartj/ehw564 28104790

[B3] Association E. Association A. C. Alliance E. W. C. (2022). Atrial fibrillation: current understanding and treatment recommendations (2021). Chin. J. Card. Arrhyth 26 (01), 15–88. 10.3760/cma.j.cn113859-20211224-00264

[B4] Balshem H. Helfand M. Schünemann H. J. Oxman A. D. Kunz R. Brozek J. (2011). GRADE guidelines: 3. Rating the quality of evidence. J. Clin. Epidemiol. 64 (4), 401–406. 10.1016/j.jclinepi.2010.07.015 21208779

[B5] Blomström-Lundqvist C. Gizurarson S. Schwieler J. Jensen S. M. Bergfeldt L. Kennebäck G. (2019). Effect of catheter ablation vs antiarrhythmic medication on quality of life in patients with atrial fibrillation: the CAPTAF randomized clinical trial. JAMA 321 (11), 1059–1068. 10.1001/jama.2019.0335 30874754 PMC6439911

[B6] Brundel B. Ai X. Hills M. T. Kuipers M. F. Lip G. Y. H. de Groot N. M. S. (2022). Atrial fibrillation. Nat. Rev. Dis. Prim. 8 (1), 21. 10.1038/s41572-022-00347-9 35393446

[B7] Chen L. Y. Ribeiro A. L. P. Platonov P. G. Cygankiewicz I. Soliman E. Z. Gorenek B. (2022). P wave parameters and indices: a critical appraisal of clinical utility, challenges, and future research-a consensus document endorsed by the international society of electrocardiology and the international society for holter and noninvasive electrocardiology. Circ. Arrhythm. Electrophysiol. 15 (4), e010435. 10.1161/circep.121.010435 35333097 PMC9070127

[B8] Chinese Society of Cardiology C. M. A. Engineering A. B. o.C. S. o.B. (2023). Chinese guidelines for the diagnosis and treatment of atrial fibrillation. Chin. J. Cardiol. 51 (6), 572–618. 10.3760/cma.j.cn112148-20230416-00221 37312479

[B9] Chung M. K. Refaat M. Shen W. K. Kutyifa V. Cha Y. M. Di Biase L. (2020). Atrial fibrillation: JACC council perspectives. J. Am. Coll. Cardiol. 75 (14), 1689–1713. 10.1016/j.jacc.2020.02.025 32273035

[B10] Crowley R. Chieng D. Sugumar H. Ling L. H. Segan L. William J. (2024). Catheter ablation for persistent atrial fibrillation: patterns of recurrence and impact on quality of life and health care utilization. Eur. Heart J. 45 (29), 2604–2616. 10.1093/eurheartj/ehae291 38759110

[B11] Du C. L. (2013). Effect of Shensong Yangxin Capsule on p-wave dispersion and NT-proBNP in patients with persistent atrial fibrillation. Chin. J. Prac. Med. 40 (27), 48–50. 10.3760/cma.j.issn.1674-4756.2013.20.021

[B12] Froehlich L. Meyre P. Aeschbacher S. Blum S. Djokic D. Kuehne M. (2019). Left atrial dimension and cardiovascular outcomes in patients with and without atrial fibrillation: a systematic review and meta-analysis. Heart 105 (24), 1884–1891. 10.1136/heartjnl-2019-315174 31422362

[B13] Ge T. Zou R. J. Zhang M. Hu J. L. He K. Y. Li G. M. (2025). Natural products alleviate atrial fibrillation by modulating mitochondrial quality control. Phytomedicine 140, 156555. 10.1016/j.phymed.2025.156555 40056631

[B14] He H. Ping J. Jiang C. H. Liao H. R. (2018). Clinical efficacy of Shensong Yangxin Capsules combined with valsartan capsules in patients with hypertension complicated by persistent atrial fibrillation. Chin. Tradit. Pat. Med. 40 (11), 2403–2407. 10.3969/j.issn.1001-1528.2018.11.009

[B15] He W. F. Xue C. Zheng J. K. Shuai Z. Yue R. C. (2024). Effects of Shensong Yangxin Capsule combined with sacubitril/valsartan on hs-CRP, BNP, AngⅡ and cardiac function in the treatment of paroxysmal atrial fibrillation complicated with chronic heart failure. Chin. Arch. Tradi Chin. Med. 42 (06), 95–98. 10.13193/j.issn.1673-7717.2024.06.018

[B16] Huang H. Liu Y. Shuai W. Jiang C. Zhang M. Qu X. (2024). Atrial tachyarrhythmia prevention by Shensong Yangxin after catheter ablation for persistent atrial fibrillation: the SS-AFRF trial. Eur. Heart J. 45 (40), 4305–4314. 10.1093/eurheartj/ehae532 39178138 PMC11491151

[B17] Jiang C. Wang X. Dang S. Wang X. Deng Q. Hu J. (2021). Chinese medicine Shensong Yangxin Capsule ameliorates myocardial microcirculation dysfunction in rabbits with chronic myocardial infarction. Chin. J. Integr. Med. 27 (1), 24–30. 10.1007/s11655-018-2578-1 30656600

[B18] Jin J. (2017). Observation on the efficacy of Shensong Yangxin Capsule combined with amiodarone in cardioverting early persistent atrial fibrillation after radiofrequency ablation. Mod. J. Integr. Tradi Chin. West Med. 26 (25), 2773–2775. 10.3969/j.issn.1008-8849.2017.25.012

[B19] Joglar J. A. Chung M. K. Armbruster A. L. Benjamin E. J. Chyou J. Y. Cronin E. M. (2024). 2023 ACC/AHA/ACCP/HRS guideline for the diagnosis and management of atrial fibrillation: a report of the American College of Cardiology/American Heart Association Joint Committee on clinical practice guidelines. Circulation 149 (1), e1–e156. 10.1161/cir.0000000000001193 38033089 PMC11095842

[B20] Karanikola A. E. Tzortzi M. Kordalis A. Doundoulakis I. Antoniou C. K. Laina A. (2025). Clinical, electrocardiographic and echocardiographic predictors of atrial fibrillation recurrence after pulmonary vein isolation. J. Clin. Med. 14 (3), 809. 10.3390/jcm14030809 39941478 PMC11818469

[B21] Kornej J. Börschel C. S. Benjamin E. J. Schnabel R. B. (2020). Epidemiology of atrial fibrillation in the 21st century: novel methods and new insights. Circ. Res. 127 (1), 4–20. 10.1161/circresaha.120.316340 32716709 PMC7577553

[B22] Li H. Song X. Liang Y. Bai X. Liu-Huo W. S. Tang C. (2022). Global, regional, and national burden of disease study of atrial fibrillation/flutter, 1990-2019: results from a global burden of disease study, 2019. BMC Public Health 22 (1), 2015. 10.1186/s12889-022-14403-2 36329400 PMC9632152

[B23] Li W. N. Cheng X. Z. Zhu G. H. Hu Y. Wang Y. H. Niu Y. Y. (2024). A review of chemotherapeutic drugs-induced arrhythmia and potential intervention with traditional Chinese medicines. Front. Pharmacol. 15, 1340855. 10.3389/fphar.2024.1340855 38572424 PMC10987752

[B24] Li X. M. (2020). Observation on the efficacy of Shensong Yangxin Capsules combined with valsartan capsules in the treatment of hypertension complicated by persistent atrial fibrillation. J. Med. Theor. Prac. 33 (8), 1258–1259. 10.19381/j.issn.1001-7585.2020.08.021

[B25] Luo D. Wan X. Liu J. Tong T. (2018). Optimally estimating the sample mean from the sample size, median, mid-range, and/or mid-quartile range. Stat. Methods Med. Res. 27 (6), 1785–1805. 10.1177/0962280216669183 27683581

[B26] Ma J. Yin C. Ma S. Qiu H. Zheng C. Chen Q. (2018). Shensong Yangxin capsule reduces atrial fibrillation susceptibility by inhibiting atrial fibrosis in rats with post-myocardial infarction heart failure. Drug Des. Devel Ther. 12, 3407–3418. 10.2147/dddt.S182834 PMC6186904 30349194

[B27] Marks D. Ho R. Then R. Weinstock J. L. Teklemariam E. Kakadia B. (2021). Real-world experience with implantable loop recorder monitoring to detect subclinical atrial fibrillation in patients with cryptogenic stroke: the value of p wave dispersion in predicting arrhythmia occurrence. Int. J. Cardiol. 327, 86–92. 10.1016/j.ijcard.2020.11.019 33186666

[B28] Medicines S. P. T. f.t.C. A. G. f.t.T. o.D. D. w.C. P. (2021). Clinical application guidelines for the treatment of coronary heart disease with Chinese patent medicines (2020). Chin. J. Integr. Tradi West Med. 41 (04), 391–417. 10.7661/j.cjim.20210201.100

[B29] Menichelli D. Sciacqua A. Cangemi R. Andreozzi P. Del Sole F. Violi F. (2021). Atrial fibrillation pattern, left atrial diameter and risk of cardiovascular events and mortality. A prospective multicenter cohort study. Int. J. Clin. Pract. 75 (3), e13771. 10.1111/ijcp.13771 33078565

[B30] Packer D. L. Mark D. B. Robb R. A. Monahan K. H. Bahnson T. D. Poole J. E. (2019). Effect of catheter ablation vs antiarrhythmic drug therapy on mortality, stroke, bleeding, and cardiac arrest among patients with atrial fibrillation: the CABANA randomized clinical trial. JAMA 321 (13), 1261–1274. 10.1001/jama.2019.0693 30874766 PMC6450284

[B31] Page M. J. McKenzie J. E. Bossuyt P. M. Boutron I. Hoffmann T. C. Mulrow C. D. (2021). The PRISMA 2020 statement: an updated guideline for reporting systematic reviews. BMJ 372, n71. 10.1136/bmj.n71 33782057 PMC8005924

[B32] Schoene K. Sepehri Shamloo A. Sommer P. Jahnke C. Paetsch I. Hindricks G. (2019). Natural course of acquired pulmonary vein stenosis after radiofrequency ablation for atrial fibrillation-Is routine follow-up imaging indicated or not? J. Cardiovasc Electrophysiol. 30 (10), 1786–1791. 10.1111/jce.14042 31231906

[B33] Shen N. Li X. Zhou T. Bilal M. U. Du N. Hu Y. (2014). Shensong Yangxin Capsule prevents diabetic myocardial fibrosis by inhibiting TGF-β1/Smad signaling. J. Ethnopharmacol. 157, 161–170. 10.1016/j.jep.2014.09.035 25267579

[B34] Steinberg B. A. Hellkamp A. S. Lokhnygina Y. Patel M. R. Breithardt G. Hankey G. J. (2015). Higher risk of death and stroke in patients with persistent vs. paroxysmal atrial fibrillation: results from the ROCKET-AF Trial. Eur. Heart J. 36 (5), 288–296. 10.1093/eurheartj/ehu359 25209598 PMC4313363

[B35] Sterne J. A. C. Savović J. Page M. J. Elbers R. G. Blencowe N. S. Boutron I. (2019). RoB 2: a revised tool for assessing risk of bias in randomised trials. BMJ 366, l4898. 10.1136/bmj.l4898 31462531

[B36] Tuo T. Zhang X. Cheng Y. L. D M. L. Zhao Q. (2020). Effects of Shensong Yangxin Capsule with valsartan on sinus rhythm maintenance and left atrial function when treating paroxysmal atrial fibrillation. Chin. Arch. Tradi Chin. Med. 38 (02), 251–254. 10.13193/j.issn.1673-7717.2020.02.062

[B37] Turagam M. K. Neuzil P. Schmidt B. Reichlin T. Neven K. Metzner A. (2023). Safety and effectiveness of pulsed field ablation to treat atrial fibrillation: one-year outcomes from the MANIFEST-PF registry. Circulation 148 (1), 35–46. 10.1161/circulationaha.123.064959 37199171

[B38] Van Gelder I. C. Rienstra M. Bunting K. V. Casado-Arroyo R. Caso V. Crijns H. (2024). 2024 ESC Guidelines for the management of atrial fibrillation developed in collaboration with the European Association for Cardio-Thoracic Surgery (EACTS). Eur. Heart J. 45 (36), 3314–3414. 10.1093/eurheartj/ehae176 39210723

[B39] Wan X. Wang W. Liu J. Tong T. (2014). Estimating the sample mean and standard deviation from the sample size, median, range and/or interquartile range. BMC Med. Res. Methodol. 14, 135. 10.1186/1471-2288-14-135 25524443 PMC4383202

[B40] Yang H. J. Kong B. Shuai W. Zhang J. J. Huang H. (2020). Shensong Yangxin protects against metabolic syndrome-induced ventricular arrhythmias by inhibiting electrical remodeling. Front. Pharmacol. 11, 993. 10.3389/fphar.2020.00993 32733242 PMC7363804

[B41] Yang H. J. Kong B. Shuai W. Zhang J. J. Huang H. (2022). Shensong Yangxin attenuates metabolic syndrome-induced atrial fibrillation via inhibition of ferroportin-mediated intracellular iron overload. Phytomedicine 101, 154086. 10.1016/j.phymed.2022.154086 35421806

[B42] Yu Z. X. Liu D. Y. (2014). Effect of Shensong Yangxin Capsule on plasma high-sensitivity C-reactive protein and N-terminal pro-brain natriuretic peptide levels after cardioversion in patients with persistent atrial fibrillation. Chin. J. Prim. Med. Pharm. 11 (21), 1670–1672. 10.3760/cma.j.issn.1008-6706.2014.11.030

[B43] Zhang G. W. Chen S. Q. Zhang M. H. Zuo Y. Y. Chen Y. Li Y. (2023a). Effect of Shensong Yangxin Capsules on left ventricular function and quality of life in patients with persistent atrial fibrillation after radiofrequency ablation. J. Diffic. Compl Cas. 22 (01), 8–13+20. 10.3969/j.issn.1671-6450.2023.01.002

[B44] Zhang J. Li H. Wang D. Gu J. Hou Y. Wu Y. (2023b). Shensong Yangxin Capsule reduces the susceptibility of arrhythmia in db/db mice via inhibiting the inflammatory response induced by endothelium dysfunction. Drug Des. Devel Ther. 17, 313–330. 10.2147/dddt.S392328 PMC9912345 36776448

[B45] Zhang M. Z. Ding B. H. Lin Q. (2021). Clinical practice guideline of Chinese medicine for acute myocardial infarction. Chin. J. Tradi Chin. Med. Pharm. 36 (07), 4119–4127.

[B46] Zhao H. Y. Zhang S. D. Zhang K. Wang X. Zhao Q. Y. Zhang S. J. (2017). Effect of Shensong Yangxin on the progression of paroxysmal atrial fibrillation is correlated with regulation of autonomic nerve activity. Chin. Med. J. Engl. 130 (2), 171–178. 10.4103/0366-6999.197997 28091409 PMC5282674

[B47] Zhao Y. Gao F. Zhang Y. Wang H. Zhu J. Chang L. (2016). Shensong Yangxin capsules prevent ischemic arrhythmias by prolonging action potentials and alleviating Ca2+ overload. Mol. Med. Rep. 13 (6), 5185–5192. 10.3892/mmr.2016.5203 27122298

[B48] Zhou J. N. Zhong D. Wang F. Zuo J. (2019). Adjuvant treatment of 60 cases of early recurrence after radiofrequency ablation for atrial fibrillation with Shensong Yangxin Capsules. Her. Med. 38 (08), 1022–1025. 10.3870/j.issn.1004-0781.2019.08.009

[B49] Zhou L. Lu F. F. (2021). Effect of Shensong Yangxin Capsules on myocardial ischemia and P-wave dispersion in patients with hypertension complicated by persistent atrial fibrillation. Int. Med. Health Guid News 27 (08), 1226–1229. 10.3760/cma.j.issn.1007-1245.2021.08.030

[B50] Zhuo C. G. Wang S. Zhang K. Hu S. Yang Y. S. Wu G. (2017). Preventive effect of Shensong Yangxin Capsule on recurrence after radiofrequency ablation for atrial fibrillation. J. Diffic. Compl Cas. 16 (03), 293–296+301. 10.3969/j.issn.1671-6450.2017.03.019

[B51] Zoni-Berisso M. Lercari F. Carazza T. Domenicucci S. (2014). Epidemiology of atrial fibrillation: European perspective. Clin. Epidemiol. 6, 213–220. 10.2147/clep.S47385 24966695 PMC4064952

