# Supplementary material for: Efficacy and safety of shensong yangxin capsules for persistent atrial fibrillation: a Systematic Review and meta-analysis of randomized controlled trials
Source: Front Pharmacol. 2025 Jun 18;16:1620340. doi: 10.3389/fphar.2025.1620340 (PMC12213544; doi:10.3389/fphar.2025.1620340)
Supplement: Supplementary file 1 [file DataSheet1.docx]

Supplementary Material

# Supplementary Tables

## Table S1. The search strategies for all databases.

| **The search strategy for PubMed** | |
| --- | --- |
| **Number** | **Search terms** |
| #1 | Atrial Fibrillation[MeSH Terms] |
| #2 | (((((atrial fibrillat*[Title/Abstract]) OR (atrial flutter*[Title/Abstract])) OR (atrium fibrillat*[Title/Abstract])) OR (atrium flutter*[Title/Abstract])) OR (auricular fibrillat*[Title/Abstract])) OR (auricular flutter*[Title/Abstract])) |
| #3 | #1 OR #2 |
| #4 | (((shen-song-yang-xin[Title/Abstract]) OR (shen song yang xin[Title/Abstract])) OR (shensongyangxin[Title/Abstract]) OR (shensong yangxin[Title/Abstract]) |
| #5 | #3 AND #4 |
| **The search strategy for Embase** | |
| **Number** | **Search terms** |
| #1 | 'atrial fibrillation'/exp |
| #2 | 'atrial fibrillat*':ab,ti OR 'atrial flutter*':ab,ti OR 'atrium fibrillat*':ab,ti OR 'atrium flutter*':ab,ti OR 'auricular fibrillat*':ab,ti OR 'auricular flutter*':ab,ti |
| #3 | #1 OR #2 |
| #4 | 'shen-song-yang-xin':ab,ti OR 'shen song yang xin':ab,ti OR 'shensongyangxin':ab,ti OR 'shensong yangxin':ab,ti |
| #5 | #3 AND #4 |
| **The search strategy for Cochrane Library** | |
| **Number** | **Search terms** |
| #1 | MeSH descriptor: [Atrial Fibrillation] explode all trees |
| #2 | (atrial fibrillat*):ti,ab,kw OR (atrial flutter*):ti,ab,kw OR (atrium fibrillat*):ti,ab,kw OR (atrium flutter*):ti,ab,kw OR (auricular fibrillat*):ti,ab,kw OR (auricular flutter*):ti,ab,kw (Word variations have been searched) |
| #3 | #1 OR #2 |
| #4 | (shen-song-yang-xin):ti,ab,kw OR (shen song yang xin):ti,ab,kw OR (shensongyangxin):ti,ab,kw OR (shensong yangxin):ti,ab,kw |
| #5 | #3 AND #4 |
| **The search strategy for CNKI** | |
| (SU = '心房颤动' OR SU = '心房纤颤' OR SU = '房颤' OR SU = '心悸' OR SU = '怔忡') AND (SU= '参松养心') | |
| **The search strategy for WanFang** | |
| 主题: ("心房颤动" OR "心房纤颤" OR "房颤") and 主题: ("参松养心") | |
| **The search strategy for VIP** | |
| ((M=心房颤动 OR 心房纤颤 OR 房颤) OR (R=心房颤动 OR 心房纤颤 OR 房颤)) AND ((M=参松养心) OR (R=参松养心)) | |
| **The search strategy for CBM** | |
| #1 | "心房颤动"[常用字段:智能] OR "心房纤颤"[常用字段:智能] OR "房颤"[常用字段:智能] |
| #2 | "参松养心"[常用字段:智能] |
| #3 | #1 AND #2 |

**Table S2**. The list of excluded reports.

| **Report excluded** | **Reason** |
| --- | --- |
| Han Weihong 2006 | Lack of efficacy outcomes |
| Song Guangyao 2009 | Lack of efficacy outcomes |
| Li Fenglian 2009 | Lack of efficacy outcomes |
| Cui Yunfeng 2010 | Lack of efficacy outcomes |
| Bai Zhidong 2017 | Lack of efficacy outcomes |
| Li Guojun 2009 | Non random |
| Wang Haixia 2017 | Non random |
| Zhang Hui 2024 | Non random |

**References**

Han, W. H. (2006). Efficacy observation of Shensong Yangxin Capsules in controlling the ventricular rate of patients with persistent atrial fibrillation. *Chinese* *Journal of Difficult and Complicated Cases.* 5(04), 284-285.

Song, G. Y., and Li, D. W. (2009). Treatment of persistent atrial fibrillation with Shensong Yangxin Capsules combined with Simvastatin. *Clinical Medicine.* 5(29), 122-123.

Li, F. L., and Deng, X. J. (2009). Clinical observation on the combined treatment of persistent atrial fibrillation with Shensong Yangxin Capsules and Carvedilol. *Xinjiang Medical Journal*. 39(06), 50-52.

Cui, Y. F. (2010). Clinical Observation of 48 Cases on Shensong Yangxin Capsules in Controlling the Ventricular Rate of Persistent Atrial Fibrillation. *Chinese Journal of Modern Drug Application.* 4(13), 125-126.

Bai, Z. D., Liu, L. H., Dang, T., Qiao, S. Z., and Wang, Y. (2017). Effect of Shensong Yangxin Capsule Combined with Bisoprolol Fumarate on Anxiety or Depression in Patients with Atrial Fibrillation. *Medical Journal of National Defending Forces in Northwest China*. 38(05), 321-323.

Li, G. J., Li, S. H., Miao, Y. G., Chen, J. L., and Han, L. (2009). Observation on the Efficacy of Shensong Yangxin Capsules Combined with Digoxin in the Treatment of Persistent and Permanent Atrial Fibrillation. *Hebei Medical Journal.* 31(22), 3096-3097.

Wang, H. X., Lu, Y. H., and Liu, S. (2017). Efficacy of dabigatran etexilate combined with Shensong Yangxin Capsules in the treatment of atrial fibrillation in the elderly. *Journal of Modern Medicine & Health.* 33(05), 736-738.

Zhang, H., Zhou, L., Gao, C., and Luo, X.R. (2024). Effect of Shensong Yangxin Capsules in assisting radiofrequency ablation for persistent atrial fibrillation to convert to sinus rhythm. *Jilin Medical Journal.* 45(5), 1146-1148.

**Table S3**. The summary table of the studies included.

| **Study** | **Formulation** | **Source** | **Species** | **Quality control reported?**  **(Y/N)** | **Chemical analysis reported?**  **(Y/N)** | **A chemical characterisation of the preparation** |
| --- | --- | --- | --- | --- | --- | --- |
| Huang et al., 2024 | Shensong Yangxin Capsules | Shijiazhuang Yiling Pharmaceutical Co., Ltd | - Root of *Panax ginseng* C.A.Mey [Araliaceae; Ginseng Root] - Root of *Paeonia lactiflora* Pall [Ranunculaceae; Red Paeoniae Trichocarpae] - Root of *Ophiopogon japonicus* (Thunb.) Ker Gawl [Liliaceae; Dwarf Lilyturf Root Tuber] - Foliferous stem and branch of *Taxillus chinensis* Danse [Loranthaceae; Chinese Taxillus Herb] - Ripe pulp of *Cornus officinalis* Siebold & Zucc [Cornaceae; Common Macrocarpium Fruit] - Rhizome of *Coptis chinensis* Franch [Ranumculaceae; Chinese Goldthread Rhizome] - Root and rhizome of *Salvia miltiorrhizaSieb* Bge [Labiatae; Dan-shen Root] - Dry ripe seed of *Ziziphus jujuba* Mill [Rhamnaceae; Spine Date Seed] - Ripe fruits of *Schisandra sphenanthera* Rehder & E.H. Wilson [Magnoliaceae; Chinese Magnoliavine Fruit] - Root and rhizome of *Nardostachys grandiflora* DC [Valerianaceae; Chinese Nardostachys Root and Rhizome] - Ossature fossil of *Polypodium pseudo-amoenum* Ching [Sapindaceae;Os Draconis] - Dry female insect *Eupolyphaga sinensis* Walker [Corydiidae; Ground Beetle] | Y – Prepared according to National Drug Standards of China Food and Drug Administration (YBZ00952003-2006Z-2009) | Y-HPLC | HPLC and GC-MS  1. Ginseng Root:  Ginsenoside Rb1, Ginsenoside Rd, Ginsenoside Re, Ginsenoside Rf, Ginsenoside Rg1, Ginsenoside Rg2 (anti-inflammation, and anti-cell apoptosis);  2. Red Paeoniae Trichocarpae:  paeoniflorin (antioxidant and anti-inflammation);  3. Chinese Taxillus Herb:  quercetin (antioxidant and anti-inflammation);   1. Common Macrocarpium Fruit:   Morroniside, loganin (anti-coagulation and anti-platelet aggregation, protection of endothelial cells and cardiomyocytes);   1. Chinese Goldthread Rhizome:   ferulic acid, emodin, chlorogenic acid, protocatechualdehyde, betaine, magnoflorine, berberine hydrochloride, berberrubine, epiberberine (anti-inflammation and antioxidant, increasing coronary blood flow, reducing calcium overload in myocardial tissue, and decreasing the occurrence of arrhythmia);   1. Dan-shen Root:   salvianolic acid A, salvianolic acid B, tanshinone IIA (vasodilation, anticoagulation, antioxidant, anti-inflammation);   1. Spine Date Seed:   Spinosin (sedation, antioxidant);   1. Chinese Magnoliavine Fruit:   schisandrin A, schisandrol A, schisantherin A (antioxidant, anti-inflammation, immune regulation);  9. Chinese Nardostachys Root and Rhizome:  gallic acid, protocatechuic acid, chlorogenic acid, caffeic acid, luteolin, diosmetin, acacetin (antibacterial, anti-inflammation, antioxidant, reducing myocardial oxygen consumption, improving myocardial oxygen tolerance, and slowing down the heart rate). |
| Zhang et al., 2023a | Shensong Yangxin Capsules | Beijing Yiling Pharmaceutical Co., Ltd | - Root of *Panax ginseng* C.A.Mey [Araliaceae; Ginseng Root] - Root of *Paeonia lactiflora* Pall [Ranunculaceae; Red Paeoniae Trichocarpae] - Root of *Ophiopogon japonicus* (Thunb.) Ker Gawl [Liliaceae; Dwarf Lilyturf Root Tuber] - Foliferous stem and branch of *Taxillus chinensis* Danse [Loranthaceae; Chinese Taxillus Herb] - Ripe pulp of *Cornus officinalis* Siebold & Zucc [Cornaceae; Common Macrocarpium Fruit] - Rhizome of *Coptis chinensis* Franch [Ranumculaceae; Chinese Goldthread Rhizome] - Root and rhizome of *Salvia miltiorrhizaSieb* Bge [Labiatae; Dan-shen Root] - Dry ripe seed of *Ziziphus jujuba* Mill [Rhamnaceae; Spine Date Seed] - Ripe fruits of *Schisandra sphenanthera* Rehder & E.H. Wilson [Magnoliaceae; Chinese Magnoliavine Fruit] - Root and rhizome of *Nardostachys grandiflora* DC [Valerianaceae; Chinese Nardostachys Root and Rhizome] - Ossature fossil of *Polypodium pseudo-amoenum* Ching [Sapindaceae;Os Draconis] - Dry female insect *Eupolyphaga sinensis* Walker [Corydiidae; Ground Beetle] | Y – Prepared according to National Drug Standards of China Food and Drug Administration (YBZ00952003-2006Z-2009) | Y-HPLC |  |
| Zhou and Lu, 2021 | Shensong Yangxin Capsules | Beijing Yiling Pharmaceutical Co., Ltd | - Root of *Panax ginseng* C.A.Mey [Araliaceae; Ginseng Root] - Root of *Paeonia lactiflora* Pall [Ranunculaceae; Red Paeoniae Trichocarpae] - Root of *Ophiopogon japonicus* (Thunb.) Ker Gawl [Liliaceae; Dwarf Lilyturf Root Tuber] - Foliferous stem and branch of *Taxillus chinensis* Danse [Loranthaceae; Chinese Taxillus Herb] - Ripe pulp of *Cornus officinalis* Siebold & Zucc [Cornaceae; Common Macrocarpium Fruit] - Rhizome of *Coptis chinensis* Franch [Ranumculaceae; Chinese Goldthread Rhizome] - Root and rhizome of *Salvia miltiorrhizaSieb* Bge [Labiatae; Dan-shen Root] - Dry ripe seed of *Ziziphus jujuba* Mill [Rhamnaceae; Spine Date Seed] - Ripe fruits of *Schisandra sphenanthera* Rehder & E.H. Wilson [Magnoliaceae; Chinese Magnoliavine Fruit] - Root and rhizome of *Nardostachys grandiflora* DC [Valerianaceae; Chinese Nardostachys Root and Rhizome] - Ossature fossil of *Polypodium pseudo-amoenum* Ching [Sapindaceae;Os Draconis] - Dry female insect *Eupolyphaga sinensis* Walker [Corydiidae; Ground Beetle] | Y – Prepared according to National Drug Standards of China Food and Drug Administration (YBZ00952003-2006Z-2009) | Y-HPLC |  |
| Li, 2020 | Shensong Yangxin Capsules | Beijing Yiling Pharmaceutical Co., Ltd | - Root of *Panax ginseng* C.A.Mey [Araliaceae; Ginseng Root] - Root of *Paeonia lactiflora* Pall [Ranunculaceae; Red Paeoniae Trichocarpae] - Root of *Ophiopogon japonicus* (Thunb.) Ker Gawl [Liliaceae; Dwarf Lilyturf Root Tuber] - Foliferous stem and branch of *Taxillus chinensis* Danse [Loranthaceae; Chinese Taxillus Herb] - Ripe pulp of *Cornus officinalis* Siebold & Zucc [Cornaceae; Common Macrocarpium Fruit] - Rhizome of *Coptis chinensis* Franch [Ranumculaceae; Chinese Goldthread Rhizome] - Root and rhizome of *Salvia miltiorrhizaSieb* Bge [Labiatae; Dan-shen Root] - Dry ripe seed of *Ziziphus jujuba* Mill [Rhamnaceae; Spine Date Seed] - Ripe fruits of *Schisandra sphenanthera* Rehder & E.H. Wilson [Magnoliaceae; Chinese Magnoliavine Fruit] - Root and rhizome of *Nardostachys grandiflora* DC [Valerianaceae; Chinese Nardostachys Root and Rhizome] - Ossature fossil of *Polypodium pseudo-amoenum* Ching [Sapindaceae;Os Draconis] - Dry female insect *Eupolyphaga sinensis* Walker [Corydiidae; Ground Beetle] | Y – Prepared according to National Drug Standards of China Food and Drug Administration (YBZ00952003-2006Z-2009) | Y-HPLC |  |
| Zhou et al., 2019 | Shensong Yangxin Capsules | Shijiazhuang Yiling Pharmaceutical Co., Ltd | - Root of *Panax ginseng* C.A.Mey [Araliaceae; Ginseng Root] - Root of *Paeonia lactiflora* Pall [Ranunculaceae; Red Paeoniae Trichocarpae] - Root of *Ophiopogon japonicus* (Thunb.) Ker Gawl [Liliaceae; Dwarf Lilyturf Root Tuber] - Foliferous stem and branch of *Taxillus chinensis* Danse [Loranthaceae; Chinese Taxillus Herb] - Ripe pulp of *Cornus officinalis* Siebold & Zucc [Cornaceae; Common Macrocarpium Fruit] - Rhizome of *Coptis chinensis* Franch [Ranumculaceae; Chinese Goldthread Rhizome] - Root and rhizome of *Salvia miltiorrhizaSieb* Bge [Labiatae; Dan-shen Root] - Dry ripe seed of *Ziziphus jujuba* Mill [Rhamnaceae; Spine Date Seed] - Ripe fruits of *Schisandra sphenanthera* Rehder & E.H. Wilson [Magnoliaceae; Chinese Magnoliavine Fruit] - Root and rhizome of *Nardostachys grandiflora* DC [Valerianaceae; Chinese Nardostachys Root and Rhizome] - Ossature fossil of *Polypodium pseudo-amoenum* Ching [Sapindaceae;Os Draconis] - Dry female insect *Eupolyphaga sinensis* Walker [Corydiidae; Ground Beetle] | Y – Prepared according to National Drug Standards of China Food and Drug Administration (YBZ00952003-2006Z-2009) | Y-HPLC |  |
| He et al., 2018 | Shensong Yangxin Capsules |  | - Root of *Panax ginseng* C.A.Mey [Araliaceae; Ginseng Root] - Root of *Paeonia lactiflora* Pall [Ranunculaceae; Red Paeoniae Trichocarpae] - Root of *Ophiopogon japonicus* (Thunb.) Ker Gawl [Liliaceae; Dwarf Lilyturf Root Tuber] - Foliferous stem and branch of *Taxillus chinensis* Danse [Loranthaceae; Chinese Taxillus Herb] - Ripe pulp of *Cornus officinalis* Siebold & Zucc [Cornaceae; Common Macrocarpium Fruit] - Rhizome of *Coptis chinensis* Franch [Ranumculaceae; Chinese Goldthread Rhizome] - Root and rhizome of *Salvia miltiorrhizaSieb* Bge [Labiatae; Dan-shen Root] - Dry ripe seed of *Ziziphus jujuba* Mill [Rhamnaceae; Spine Date Seed] - Ripe fruits of *Schisandra sphenanthera* Rehder & E.H. Wilson [Magnoliaceae; Chinese Magnoliavine Fruit] - Root and rhizome of *Nardostachys grandiflora* DC [Valerianaceae; Chinese Nardostachys Root and Rhizome] - Ossature fossil of *Polypodium pseudo-amoenum* Ching [Sapindaceae;Os Draconis] - Dry female insect *Eupolyphaga sinensis* Walker [Corydiidae; Ground Beetle] | Y – Prepared according to National Drug Standards of China Food and Drug Administration (YBZ00952003-2006Z-2009) | Y-HPLC |  |
| Zhuo et al., 2017 | Shensong Yangxin Capsules | Shijiazhuang Yiling Pharmaceutical Co., Ltd | - Root of *Panax ginseng* C.A.Mey [Araliaceae; Ginseng Root] - Root of *Paeonia lactiflora* Pall [Ranunculaceae; Red Paeoniae Trichocarpae] - Root of *Ophiopogon japonicus* (Thunb.) Ker Gawl [Liliaceae; Dwarf Lilyturf Root Tuber] - Foliferous stem and branch of *Taxillus chinensis* Danse [Loranthaceae; Chinese Taxillus Herb] - Ripe pulp of *Cornus officinalis* Siebold & Zucc [Cornaceae; Common Macrocarpium Fruit] - Rhizome of *Coptis chinensis* Franch [Ranumculaceae; Chinese Goldthread Rhizome] - Root and rhizome of *Salvia miltiorrhizaSieb* Bge [Labiatae; Dan-shen Root] - Dry ripe seed of *Ziziphus jujuba* Mill [Rhamnaceae; Spine Date Seed] - Ripe fruits of *Schisandra sphenanthera* Rehder & E.H. Wilson [Magnoliaceae; Chinese Magnoliavine Fruit] - Root and rhizome of *Nardostachys grandiflora* DC [Valerianaceae; Chinese Nardostachys Root and Rhizome] - Ossature fossil of *Polypodium pseudo-amoenum* Ching [Sapindaceae;Os Draconis] - Dry female insect *Eupolyphaga sinensis* Walker [Corydiidae; Ground Beetle] | Y – Prepared according to National Drug Standards of China Food and Drug Administration (YBZ00952003-2006Z-2009) | Y-HPLC |  |
| Jin, 2017 | Shensong Yangxin Capsules |  | - Root of *Panax ginseng* C.A.Mey [Araliaceae; Ginseng Root] - Root of *Paeonia lactiflora* Pall [Ranunculaceae; Red Paeoniae Trichocarpae] - Root of *Ophiopogon japonicus* (Thunb.) Ker Gawl [Liliaceae; Dwarf Lilyturf Root Tuber] - Foliferous stem and branch of *Taxillus chinensis* Danse [Loranthaceae; Chinese Taxillus Herb] - Ripe pulp of *Cornus officinalis* Siebold & Zucc [Cornaceae; Common Macrocarpium Fruit] - Rhizome of *Coptis chinensis* Franch [Ranumculaceae; Chinese Goldthread Rhizome] - Root and rhizome of *Salvia miltiorrhizaSieb* Bge [Labiatae; Dan-shen Root] - Dry ripe seed of *Ziziphus jujuba* Mill [Rhamnaceae; Spine Date Seed] - Ripe fruits of *Schisandra sphenanthera* Rehder & E.H. Wilson [Magnoliaceae; Chinese Magnoliavine Fruit] - Root and rhizome of *Nardostachys grandiflora* DC [Valerianaceae; Chinese Nardostachys Root and Rhizome] - Ossature fossil of *Polypodium pseudo-amoenum* Ching [Sapindaceae;Os Draconis] - Dry female insect *Eupolyphaga sinensis* Walker [Corydiidae; Ground Beetle] | Y – Prepared according to National Drug Standards of China Food and Drug Administration (YBZ00952003-2006Z-2009) | Y-HPLC |  |
| Yu and Liu, 2014 | Shensong Yangxin Capsules | Shijiazhuang Yiling Pharmaceutical Co., Ltd | - Root of *Panax ginseng* C.A.Mey [Araliaceae; Ginseng Root] - Root of *Paeonia lactiflora* Pall [Ranunculaceae; Red Paeoniae Trichocarpae] - Root of *Ophiopogon japonicus* (Thunb.) Ker Gawl [Liliaceae; Dwarf Lilyturf Root Tuber] - Foliferous stem and branch of *Taxillus chinensis* Danse [Loranthaceae; Chinese Taxillus Herb] - Ripe pulp of *Cornus officinalis* Siebold & Zucc [Cornaceae; Common Macrocarpium Fruit] - Rhizome of *Coptis chinensis* Franch [Ranumculaceae; Chinese Goldthread Rhizome] - Root and rhizome of *Salvia miltiorrhizaSieb* Bge [Labiatae; Dan-shen Root] - Dry ripe seed of *Ziziphus jujuba* Mill [Rhamnaceae; Spine Date Seed] - Ripe fruits of *Schisandra sphenanthera* Rehder & E.H. Wilson [Magnoliaceae; Chinese Magnoliavine Fruit] - Root and rhizome of *Nardostachys grandiflora* DC [Valerianaceae; Chinese Nardostachys Root and Rhizome] - Ossature fossil of *Polypodium pseudo-amoenum* Ching [Sapindaceae;Os Draconis] - Dry female insect *Eupolyphaga sinensis* Walker [Corydiidae; Ground Beetle] | Y – Prepared according to National Drug Standards of China Food and Drug Administration (YBZ00952003-2006Z-2009) | Y-HPLC |  |
| Du, 2013 | Shensong Yangxin Capsules | Shijiazhuang Yiling Pharmaceutical Co., Ltd | - Root of *Panax ginseng* C.A.Mey [Araliaceae; Ginseng Root] - Root of *Paeonia lactiflora* Pall [Ranunculaceae; Red Paeoniae Trichocarpae] - Root of *Ophiopogon japonicus* (Thunb.) Ker Gawl [Liliaceae; Dwarf Lilyturf Root Tuber] - Foliferous stem and branch of *Taxillus chinensis* Danse [Loranthaceae; Chinese Taxillus Herb] - Ripe pulp of *Cornus officinalis* Siebold & Zucc [Cornaceae; Common Macrocarpium Fruit] - Rhizome of *Coptis chinensis* Franch [Ranumculaceae; Chinese Goldthread Rhizome] - Root and rhizome of *Salvia miltiorrhizaSieb* Bge [Labiatae; Dan-shen Root] - Dry ripe seed of *Ziziphus jujuba* Mill [Rhamnaceae; Spine Date Seed] - Ripe fruits of *Schisandra sphenanthera* Rehder & E.H. Wilson [Magnoliaceae; Chinese Magnoliavine Fruit] - Root and rhizome of *Nardostachys grandiflora* DC [Valerianaceae; Chinese Nardostachys Root and Rhizome] - Ossature fossil of *Polypodium pseudo-amoenum* Ching [Sapindaceae;Os Draconis] - Dry female insect *Eupolyphaga sinensis* Walker [Corydiidae; Ground Beetle] | Y – Prepared according to National Drug Standards of China Food and Drug Administration (YBZ00952003-2006Z-2009) | Y-HPLC |  |

# Supplementary Figures


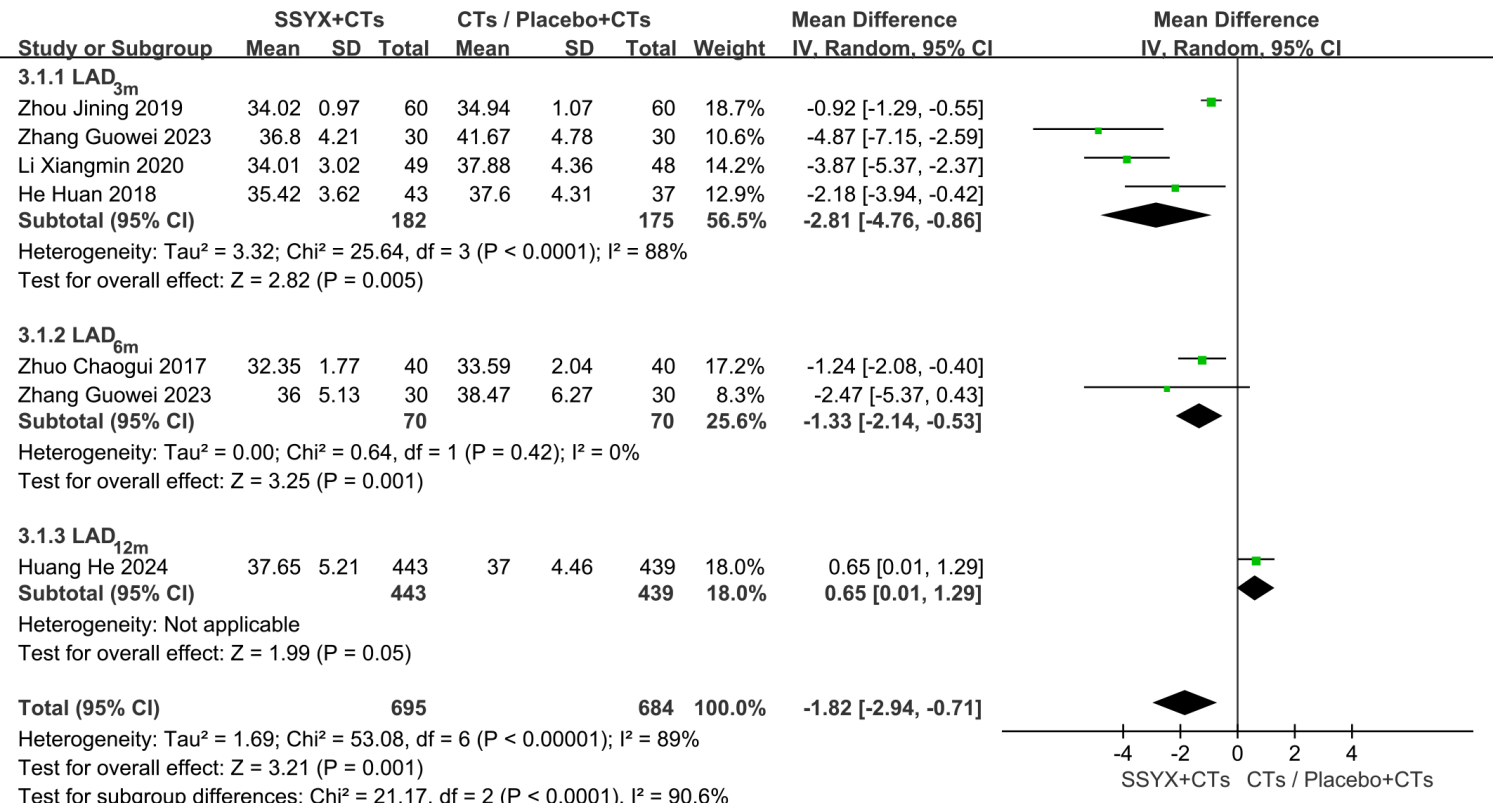


**Supplementary Figure S1**. LAD: SSYX plus CTs vs. Placebo plus CTs / CTs.


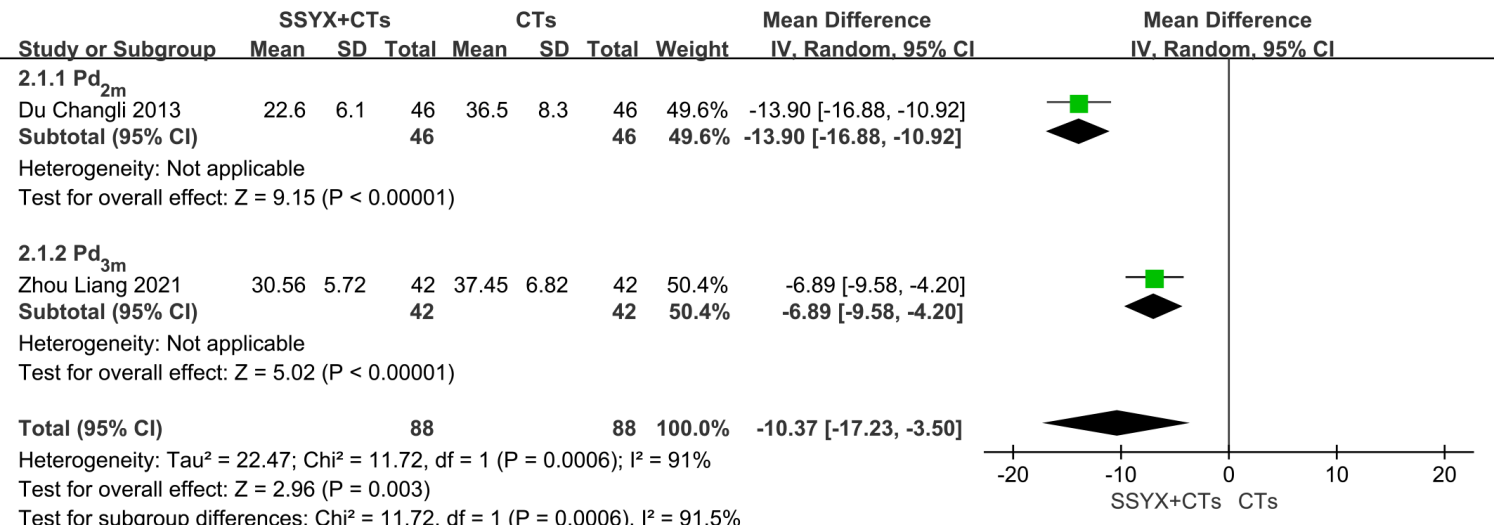


**Supplementary Figure S2**. Pd: SSYX plus CTs vs. CTs.


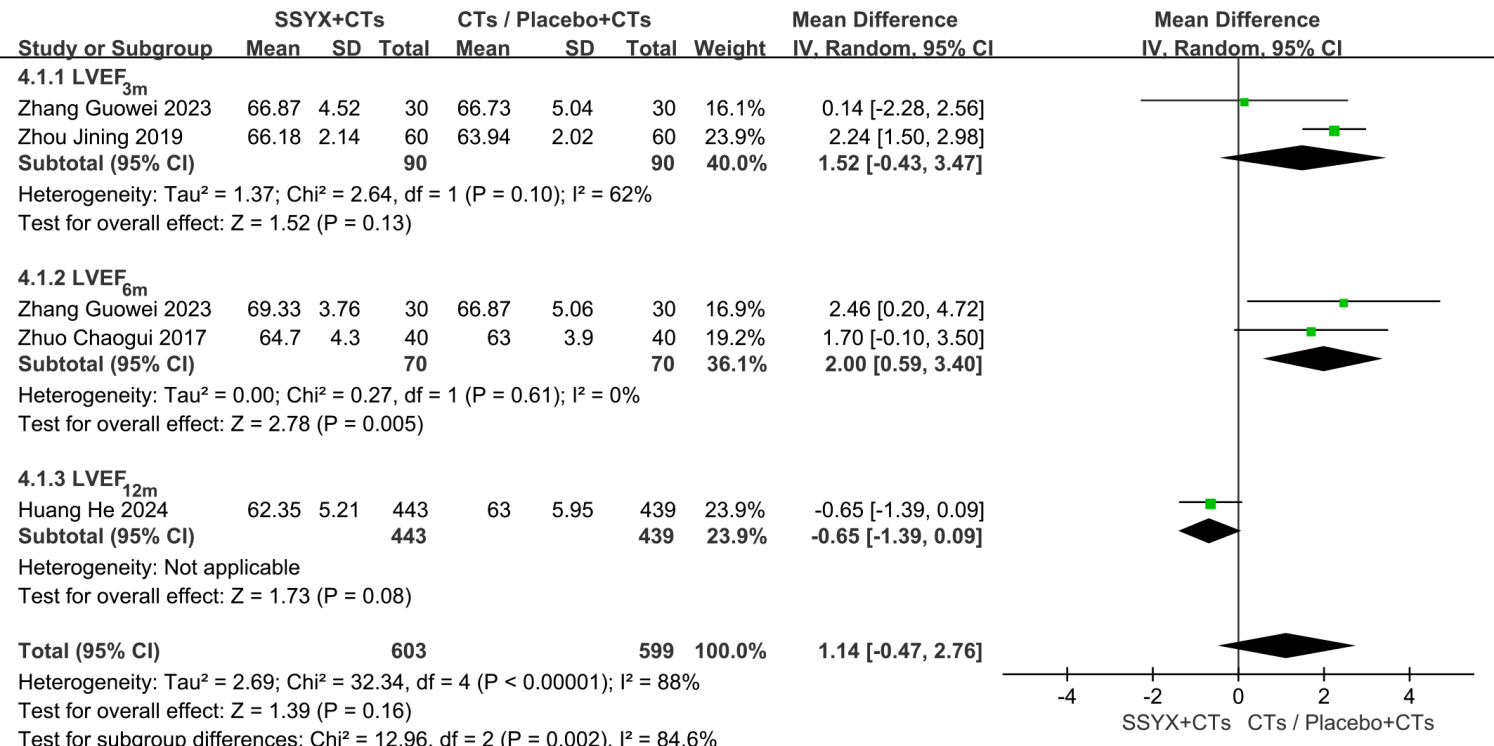


**Supplementary Figure S3.** LVEF: SSYX plus CTs vs. Placebo plus CTs / CTs.


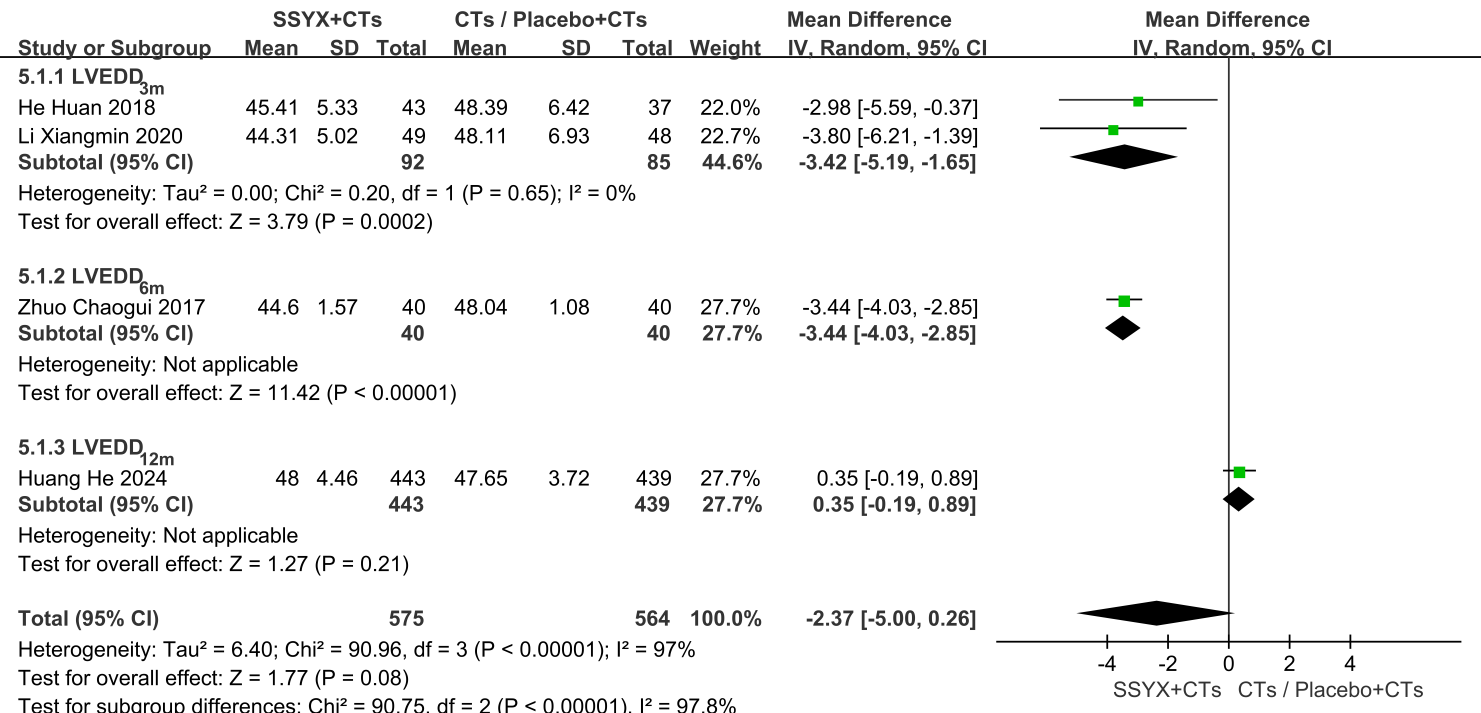


**Supplementary Figure S4**. LVEDD: SSYX plus CTs vs. Placebo plus CTs / CTs.


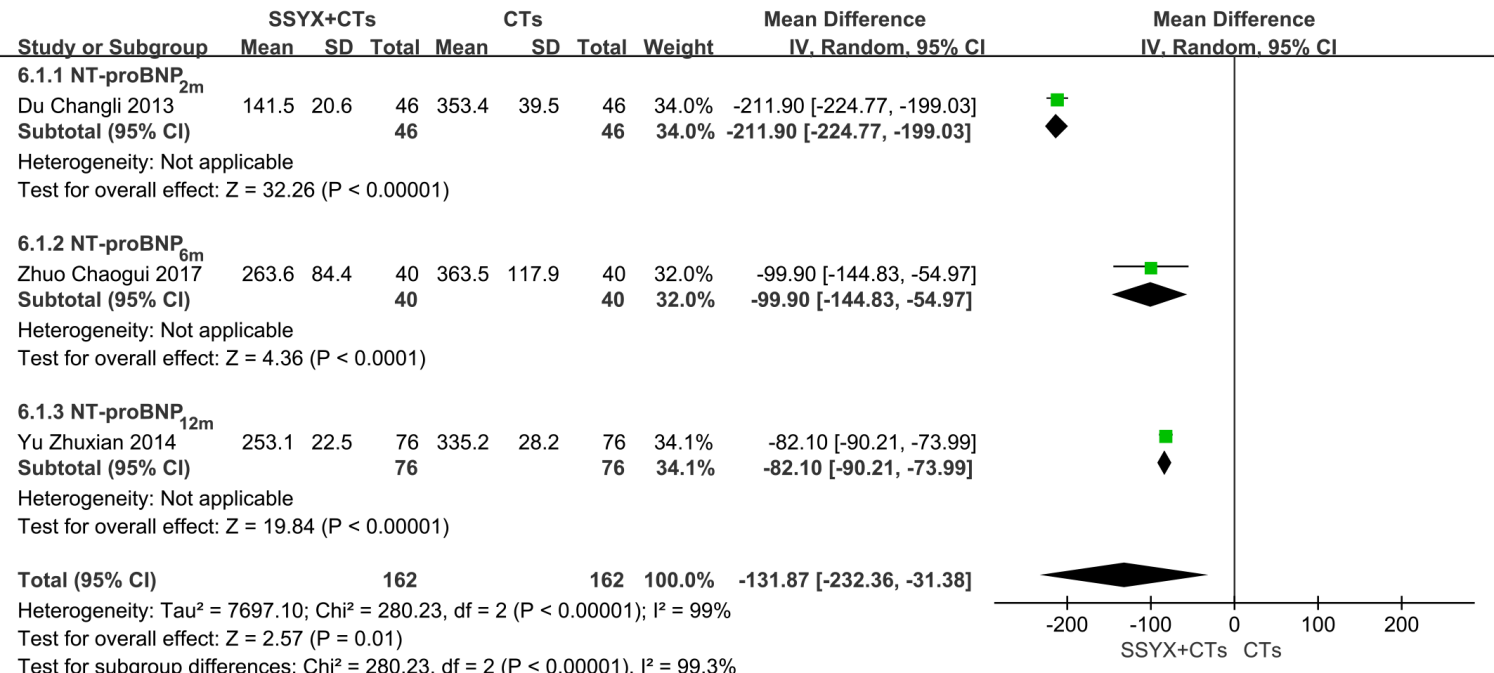


**Supplementary Figure S5**. NT-proBNP: SSYX plus CTs vs. CTs.


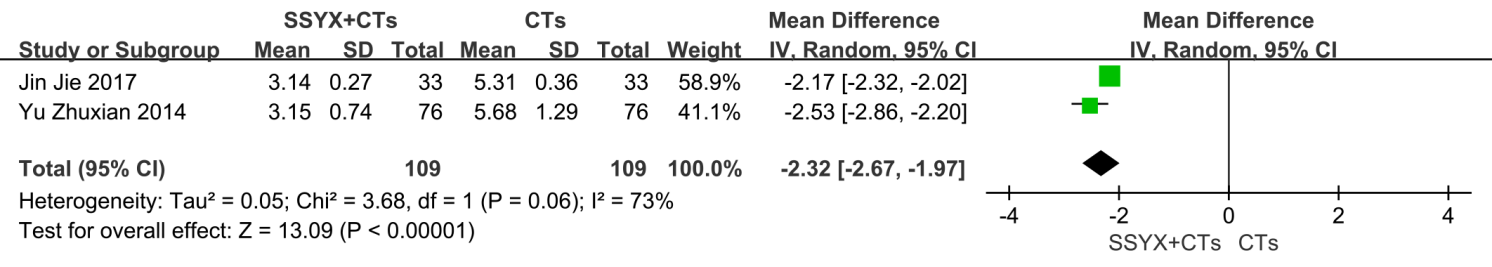


**Supplementary Figure S6**. hs-CRP: SSYX plus CTs vs. CTs.
